# Improving trial questionnaire response rates using behaviour change theory

**DOI:** 10.1186/1745-6215-16-S2-P92

**Published:** 2015-11-16

**Authors:** Anne Duncan, Debbie Bonetti, Jan Clarkson, Craig Ramsay

**Affiliations:** 1 University of Aberdeen, Aberdeen, UK; 2 University of Dundee, Dundee, UK

## 

A number of options to improve response rates to postal questionnaires in trials have been reported, but the evidence to support the success of some of these approaches is limited. Robust, linked, evaluation of novel strategies is needed to build a coherent evidence base. The response rate to the first of three annual postal questionnaires was lower than expected in the NIHR HTA funded IQuaD (Improving the Quality of Dentistry) Trial, a randomised controlled trial based in dental primary care. The Theoretical Domains Framework was used to design a novel intervention to improve the response rate for subsequent questionnaires. Strategies known to influence salient domains for the target behaviour (returning the trial questionnaire) feasible to operationalise in a letter format (e.g. goal setting, persuasion) were incorporated into a cover letter. A randomised sub study within the IQuaD trial showed that the return rate was significantly higher in the group who received the theoretically informed letter than the group who received the original cover letter, [73.0% vs 66.8%, difference +6.2%, 95% CI (+1.0% to +11.4%)]. However a continuing decline in response rate at subsequent follow up, despite the amended cover letter being used, led to the development of another randomised nested sub study to investigate the effectiveness of a further theoretically informed intervention, similarly designed, in the form of a newsletter. Results from these two nested sub studies will be presented to illustrate how using psychological behaviour change theory can improve the response rate to annual questionnaires.

